# Identification and Characterization of Approved Drugs and Drug-Like Compounds as Covalent *Escherichia coli* ClpP Inhibitors

**DOI:** 10.3390/ijms20112686

**Published:** 2019-05-31

**Authors:** Elisa Sassetti, Cristina Durante Cruz, Päivi Tammela, Mathias Winterhalter, Koen Augustyns, Philip Gribbon, Björn Windshügel

**Affiliations:** 1 Fraunhofer Institute for Molecular Biology and Applied Ecology IME, ScreeningPort, 22525 Hamburg, Germany; eli.sassetti@gmail.com (E.S.); philip.gribbon@ime.fraunhofer.de (P.G.); 2 Drug Research Program, Division of Pharmaceutical Biosciences, University of Helsinki, FI-00014 Helsinki, Finland; cristina.durantecruz@helsinki.fi (C.D.C.); paivi.tammela@helsinki.fi (P.T.); 3 Department of Life Sciences and Chemistry, Jacobs University Bremen gGmbH, 28759 Bremen, Germany; m.winterhalter@jacobs-university.de; 4 Laboratory of Medicinal Chemistry, University of Antwerp, B-2610 Antwerp, Belgium; koen.augustyns@uantwerpen.be

**Keywords:** *Escherichia coli*, ClpP, high-throughput screening, inhibitor, surface plasmon resonance, covalent binding, nitric oxide stress

## Abstract

The serine protease Caseinolytic protease subunit P (ClpP) plays an important role for protein homeostasis in bacteria and contributes to various developmental processes, as well as virulence. Therefore, ClpP is considered as a potential drug target in Gram-positive and Gram-negative bacteria. In this study, we utilized a biochemical assay to screen several small molecule libraries of approved and investigational drugs for *Escherichia coli* ClpP inhibitors. The approved drugs bortezomib, cefmetazole, cisplatin, as well as the investigational drug cDPCP, and the protease inhibitor 3,4-dichloroisocoumarin (3,4-DIC) emerged as ClpP inhibitors with IC_50_ values ranging between 0.04 and 31 µM. Compound profiling of the inhibitors revealed cefmetazole and cisplatin not to inhibit the serine protease bovine α-chymotrypsin, and for cefmetazole no cytotoxicity against three human cell lines was detected. Surface plasmon resonance studies demonstrated all novel ClpP inhibitors to bind covalently to ClpP. Investigation of the potential binding mode for cefmetazole using molecular docking suggested a dual covalent binding to Ser97 and Thr168. While only the antibiotic cefmetazole demonstrated an intrinsic antibacterial effect, cDPCP clearly delayed the bacterial growth recovery time upon chemically induced nitric oxide stress in a ClpP-dependent manner.

## 1. Introduction

Antimicrobial resistance of Gram-negative bacteria has increased significantly over the last decades [1,2]. Therefore, novel approaches for antimicrobial therapies are urgently needed. Classical antibiotics inhibit protein synthesis, cell wall synthesis, depolarization of the membrane potential, folate synthesis, and DNA or RNA synthesis [3]. Caseinolytic protease subunit P (ClpP) is a well-conserved serine protease in bacteria and many eukaryotes, and absent in archaea and mollicutes [4,5]. Several studies have shown that 80% of cellular proteolysis in bacteria is dependent on the proteases ClpP and La (Lon) [6], thus suggesting a major role of ClpP in bacterial homeostasis and pathogenesis [6,7].

ClpP is a tetradecameric protein composed of two heptameric rings, which are assembled back-to-back, resulting in the formation of a proteolytic chamber which is connected to the cytoplasm via two axial channels [8]. The peptidase activity of ClpP results in peptides of seven to eight residues in length [9]. Members of the AAA+ superfamily (ATPases associated with diverse cellular activities), such as ClpX and ClpA in *E. coli*, bind to the two axial channels of tetradecameric ClpP and initiate substrate recognition, unfolding, and threading of the unfolded protein chain into the proteolytic chamber of ClpP [10,11,12].

To date, about 70 proteins have been identified as substrates for *E. coli* ClpP-mediated proteolytic cleavage [13]. In *E. coli*, ClpP is involved in metabolism, damage repair, cell division, transcriptional regulation, and stress response [14]. In other bacteria, ClpP contributes to oxidative stress response (*Streptococcus pneumoniae*) [15], virulence (*Staphylococcus aureus*) [16,17], biofilm formation and motility (*Pseudomonas aeruginosa*) [18]. It has also been shown that ClpP is essential for macrophage infection (*Listeria monocytogenes*) [19]. Impaired virulence and reduced translocation of effector proteins have been observed for clpP-deficient *Legionella pneumophila* [20]. Due to its wide range of relevant functions, ClpP is considered as a highly promising target for antibiotics [7]. Recently, differences in growth curves between wild-type (WT) and clpP-deficient *E. coli* strains under nitric oxide stress conditions suggested that targeting ClpP also in Gram-negative bacteria might represent a promising therapeutic approach [14].

Several small molecules have been described to inhibit ClpP activity, including phenyl esters, boronates, AV145 and the covalently binding benzyloxycarbonyl-leucyl-tyrosine chloromethyl ketone (Z-LY-CMK) [21,22,23]. Additionally, several β-lactones have been shown as active against various ClpPs of pathogenic and nonpathogenic bacterial strains [7]. Recently, we have reported α-amino diphenyl phosphonates as potent inhibitors of ClpP in *E. coli* [24]. Besides inhibitors, enzyme activators have also been described that activate ClpPs by preventing ClpA or ClpX binding to ClpP, thereby resulting in uncontrolled proteolysis by the enzyme [8].

In this study, we aimed to identify novel small molecules inhibiting *E. coli* ClpP. For this purpose, we screened three small molecule libraries containing approved and investigational drugs using a high-throughput biochemical assay. Validated ClpP inhibitors were further characterized for enzyme selectivity, cell toxicity, protein binding, and antimicrobial properties.

## 2. Results

The small molecule libraries SCREEN-WELL^®^ FDA approved drug library version 2 (774 compounds), LOPAC^®1280^ (1280 compounds) and a set of matrix metalloproteinase (MMP) inhibitors (329 compounds) were screened for *E. coli* ClpP inhibition. DMSO and the already known ClpP inhibitor Z-LY-CMK were used as negative or positive control, respectively. In order to assess the validity of the screening campaign, the Z’ value was calculated for each microtiter plate [25]. For all screened plates, Z’ was at least 0.6 (Figure S1), thus indicating an acceptable assay performance [26]. The primary screen resulted in 24 compounds inhibiting ClpP by >70% of which six compounds showed >90% enzyme inhibition: cisplatin, cDPCP, bortezomib, 3,4-dichloroisocoumarin (3,4-DIC), cefmetazole, and guanabenz (Table S1).

**Figure 1:**
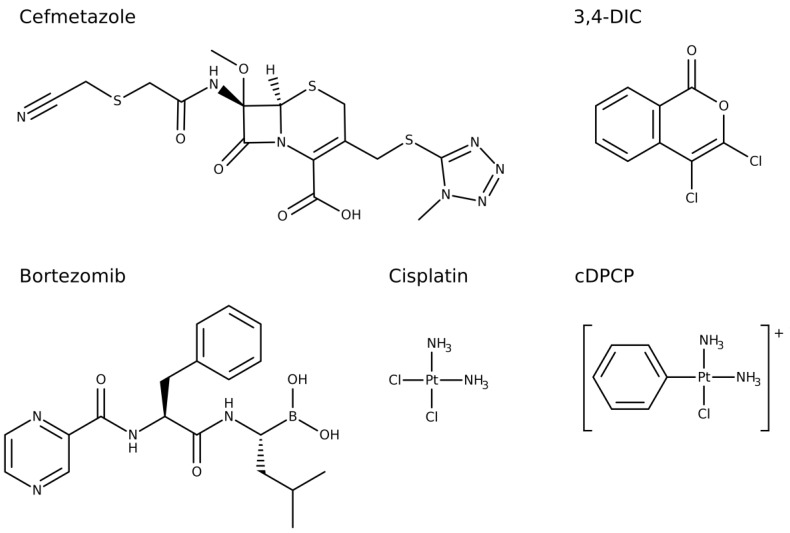
Structural formula of the five ClpP inhibitors identified in this study.

Hit validation was carried out by testing the six most potent compounds in dose-response. This confirmed five primary hits (Figure 1) as ClpP inhibitors with IC_50_ values ranging from 0.04 to 31.0 µM (Figure S2, Table 1). The confidence interval varied between 0.02 and 44.8. Only guanabenz did not confirm as ClpP inhibitor. With an IC_50_ of 0.04 μM, bortezomib emerged as the most potent compound in this study. For comparison, cefmetazole and 3,4-DIC exhibited a more than 100-fold lower potency. Cisplatin and cDPCP proved to be the least potent ClpP inhibitors. None of the other cephems and penems included in the screened compound libraries showed a ClpP inhibition exceeding 70% (Table S2). The positive control Z-LY-CMK revealed an IC_50_ value above 10 μM.

As a preliminary assessment of serine protease selectivity, we investigated the five novel ClpP inhibitors for any potential inhibition of bovine α-chymotrypsin. At 200 µM compound concentration, bortezomib, 3,4-DIC, as well as the known ClpP inhibitor Z-LY-CMK completely inhibited α-chymotrypsin and also cDPCP showed a potent enzyme inhibition of almost 85% (Table 1). In contrast, neither cefmetazole nor cisplatin affected the bovine α-chymotrypsin enzymatic activity at all.

Subsequently, the cytotoxic potential of the novel ClpP inhibitors and Z-LY-CMK against three human cell lines HTC-116 (colon), MCF-7 (breast), and A549 (lung) was assessed using a luminescent cell viability assay. Here, bortezomib revealed as highly toxic against all tested cell lines, even at the lowest tested concentration of 3.4 nM (Table 2). The already known ClpP inhibitor Z-LY-CMK emerged as highly toxic against all cell lines as well. Compared to their ClpP inhibitory potency, cDPCP, 3,4-DIC, and cisplatin also proved as toxic against all tested cell lines, as the ratio of IC_50_ values obtained for cell viability and ClpP inhibition was small (<10), except for cisplatin tested against HTC-116 cells (~29). In contrast, cefmetazole did not show any relevant cytotoxicity as the ratio was between 74 and 1597.

**Figure 2:**
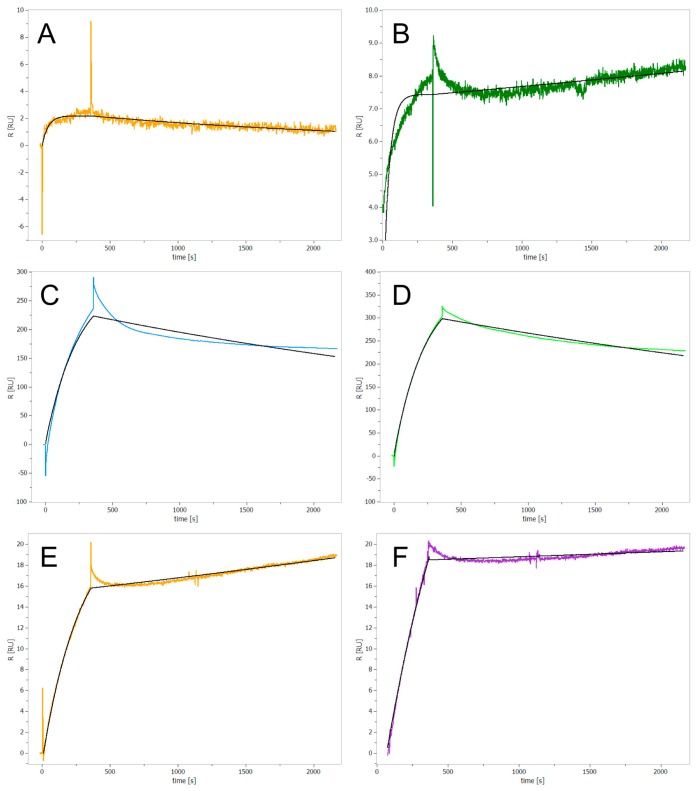
SPR sensorgrams of the ClpP inhibitors at 80 μM concentration. Each compound was injected over 7 min, followed by 30 min of dissociation time. (**A**) Bortezomib, (**B**) Cefmetazole, (**C**) cDPCP, (**D**) Cisplatin, (**E**) 3,4-DIC, and (**F**) Z-LY-CMK (covalent binder, control). The fitting curves of the SPR sensorgrams (black lines) were obtained using a 1:1 Langmuir interaction model.

In a next step, we assessed whether the identified inhibitors bind covalently or non-covalently to ClpP using surface plasmon resonance (SPR) spectroscopy. Test compounds were injected into the flow cell at 80 µM concentration. As the SPR signal did not return to baseline after 30 min, all novel ClpP inhibitors were expected to irreversibly bind to the enzyme (Figure 2A–E). While the already known covalent inhibitor Z-LY-CMK confirmed as an irreversibly binding compound (Figure 2F), caffeine, used as a non-binding control, did not show any interaction with ClpP (Figure S3). Measurements with bortezomib revealed a low change of absolute resonance unit (RU) (Figure 2A), even though this compound exhibits a nanomolar IC_50_ value in the enzymatic assay. This effect could be due to partial dissociation effects or induction of intramolecular conformational changes upon binding. For cisplatin and cDPCP we obtained high RU values, indicating unspecific binding of both compounds to the protein (Figure 2C,D).

**Figure 3:**
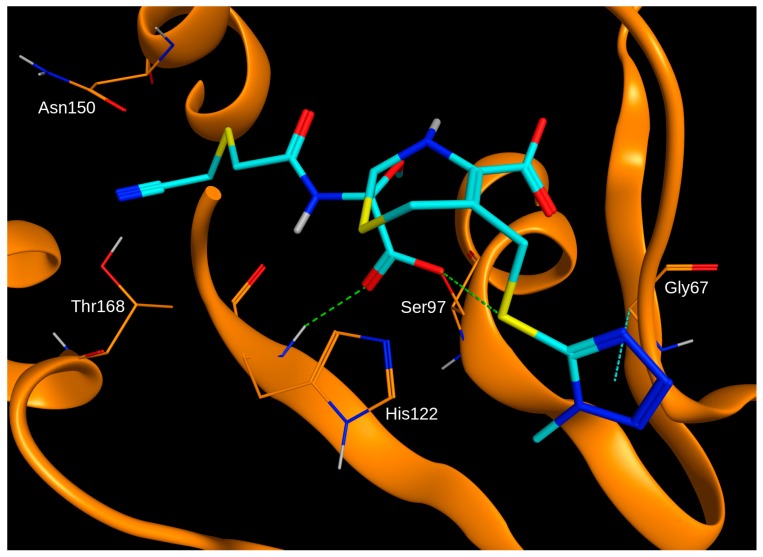
Binding mode of cefmetazole (carbon atoms in cyan) in ClpP obtained by molecular docking into Protein Data Bank (PDB) ID 2FZS using GOLD with the compound covalently attached to the hydroxyl oxygen of Ser97. Hydrogen bonds and C–H–pi interactions are indicated as green and cyan dashed lines, respectively.

In order to evaluate the potential binding mode of cefmetazole in the active site of ClpP, the compound was docked into the X-ray crystal structure of the enzyme (Protein Data Bank ID 2FZS). Due to the experimentally-confirmed irreversible binding to ClpP, cefmetazole was docked with the β-lactam ring in the open conformation and covalently bound to Ser97 of the catalytic triad, which is the most frequent covalent interaction point for penem-class antibiotics [27]. The binding mode of the top-ranked docking pose within the active site showed several hydrogen bonds shared with the protein (Figure 3). Notably, the potentially reactive nitrile moiety is placed in close proximity to Thr168.

In a next step, the compounds were tested for any intrinsic antimicrobial activity against wild-type *E. coli* BW25-113 and the isogenic ClpP-deficient strain *E. coli* JW0427 (Δ*clpP*) at a concentration of 100 μM in rich Mueller Hinton broth medium (MHB). Only the antibiotic cefmetazole inhibited growth of both strains at 100 μM concentration (Table 3), while all other compounds did not affect bacterial growth. In addition, the compounds were tested in *E. coli* JW5503 (Δ*tolC*), an efflux pump-defective strain. Except cefmetazole, none of the compounds showed improved antibacterial activity. The fact that cefmetazole completely inhibited bacterial growth of the tested strains is consistent with its role as an antibiotic; therefore, we excluded the compound from further antimicrobial investigations in this study. Next, bortezomib, cisplatin, cDPCP, and 3,4-DIC were evaluated for growth inhibition of *E. coli* BW25-113 and *E. coli* JW0427 in presence of the known efflux pump inhibitor Phe-Arg β-naphthylamide (PAβN, 25 μM). Of the tested compounds, bortezomib resulted in 55% growth inhibition in the WT strain and, interestingly, 100% in the clpP-deficient mutant (Table 3).

Recently, it has been shown that clpP-deficient *E. coli* shows an impaired nitric oxide (NO•) detoxification capacity compared to WT *E. coli* after nitric oxide stress induction using DPTA NONOate, which spontaneously dissociates and thereby releases two NO• molecules per parent compound [14]. In order to investigate the effect of the novel ClpP inhibitors on the capability of bacteria to recover from nitric oxide stress, compounds were tested at 50 and 100 μM against wild-type *E. coli* BW25-113 in minimal medium M9, in the presence or absence of DPTA NONOate, respectively. The isogenic mutant *E. coli* JW0427 (Δ*clpP*) was used as positive control.

**Figure 4:**
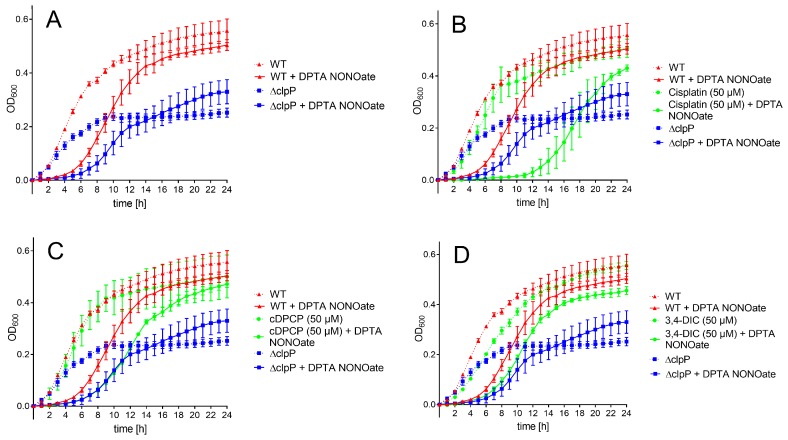
Bacterial growth curves of WT and Δ*clpP* *E. coli* strains in minimal media and in presence (solid lines) and absence (dotted lines) of DTPA NONOate (NO•). OD_600_ was measured in absence (**A**) and presence of 50 μM (**B**) cisplatin (**C**) cDPCP and (**D**) 3,4-DIC. Each value represents the mean of three independent experiment ± standard deviation.

Untreated WT and Δ*clpP* *E. coli* strain revealed similar growth within the first three hours, while the mutant *E. coli* strain continued with slower growth afterwards and did not reach the same optical density (OD_600_) level as the WT strain within 24 h (Figure 4A). Using a rich medium (Mueller Hinton broth), the clpP-deficient strain also grew slower; however, both strains reached an almost identical optical density after 24 h (Figure S4). Treatment with the NO• donor DPTA NONOate resulted in a ~3 h growth arrest for the wild-type strain and ~4 h for Δ*clpP* *E. coli*. In presence of 50 μM cisplatin, the bacterial growth was comparable to the untreated bacteria (Figure 4B). Administration of cisplatin in presence of DPTA NONOate resulted in a significantly longer growth arrest (~10 h) compared to untreated WT and mutant *E. coli* in presence of NO• stress. At a higher compound concentration (100 μM), cisplatin affected bacterial growth in the absence of NO• stress, probably due to toxicity (Figure S5A). cDPCP alone did not influence bacterial growth at both tested concentrations (Figure 4C and Figure S5B). When exposed to nitric oxide stress, cDPCP-treated WT bacteria recovered later than the stressed WT strain without compound addition. The bacterial growth in the first 11 h was identical to the growth of nitric oxide stress-exposed *E. coli* lacking ClpP (Figure 4C). At 100 μM concentration (Figure S5B), WT cell growth was clearly influenced by the compound itself. Experiments using 3,4-DIC resulted in very similar growth curves at both tested concentrations (Figure 4D and Figure S5C). We observed a small growth delay of non-stressed WT *E. coli* in the presence of the compound compared to untreated bacteria. A similar effect occurred in bacteria co-treated with DPTA NONOate. Bortezomib did not show any effect on growth curves, both in the absence and presence of nitric oxide stress (data not shown).

After 24 h, all bacteria exposed to test compounds (except cisplatin and cDPCP at 100 μM) and nitric oxide stress conditions reached an OD_600_ level comparable to WT *E. coli*, exceeding the growth levels of the mutant (Figure 4).

In order to evaluate whether the observed compound effects on growth delay are Δ*clpP*-mediated, the compounds were tested at the same conditions in clpP-deficient *E. coli*. The absence of nitric oxide stress did not result in any significant growth curve differences compared to untreated cells (Figure S6). In presence of nitric oxide stress, cisplatin showed a significantly longer growth delay compared to DPTA NONOate-exposed cells in the absence of any compound. In contrast, cDPCP and 3,4-DIC did not show any significant effect in clpP-deficient *E. coli*, and upon induction of nitric oxide stress the growth delay was virtually identical to the bacteria without compound treatment (Figure S6).

## 3. Discussion

Based on a biochemical high-throughput screen, we identified five novel and structurally distinct inhibitors of *E. coli* ClpP. These comprise two approved drugs and one investigational drug used as cancer therapeutics, an antibiotic, which is known to be active against Gram-positive and -negative bacteria, as well as a serine protease inhibitor. All ClpP inhibitors were profiled with respect to serine protease selectivity, the binding mode, the cytotoxic potential, as well as the intrinsic antimicrobial activity. In addition, we also investigated the nitric oxide (NO•) detoxification capacity of wild-type *E. coli* and a ClpP-deficient strain in the presence and absence of compounds. We confirmed the reported growth delay of the Δ*clpP* mutant as described by Robinson and Brynildsen [14], although in this study the differences between both strains were less pronounced. Moreover, our data did not show the mutant *E. coli* strain to reach the same optical density (OD_600_) level as the WT strain within 24 h, as described in the initial study [14], neither in the presence nor absence of NO• stress. Possibly, this is due to different strains used in the previous study as well as the assay setup, as shown by the growth curves in MHB medium. Alternatively, it may indicate another effect that relates to the importance of ClpP in mediating bacterial growth in conditions associated with resource shortage, (i.e., minimal medium conditions). Moreover, a strain-dependent effect cannot be excluded.

The anticancer compounds cisplatin and cDPCP revealed the highest IC_50_ values of the five novel ClpP inhibitors. Cisplatin did not show any inhibition of bovine α-chymotrypsin in this study, even at a high concentration of 200 μM. In contrast, cDPCP potently inhibited the enzyme. Cisplatin and cDPCP are metal-containing compounds used in chemotherapy. While cisplatin acts by DNA cross-linking, cDPCP makes a single mono-functional adduct with DNA due to a single chlorine atom that acts as a leaving group [28]. Due to the known mechanism of action of both compounds, the covalent interaction with ClpP is not unexpected. Considering the therapeutic area of these substances, the pronounced cytotoxicity of cDPCP and cisplatin is not surprising. Notably, both compounds showed antibacterial activity when applied in the presence of nitric oxide stress. While the effect of cisplatin on bacterial growth seems to be ClpP-independent, cDPCP appears to affect *E. coli* growth in a ClpP-mediated manner. Besides cisplatin and cDPCP, another platinum-containing compound was included in the screened compound libraries (carboplatin). However, the substance did not show any significant enzyme inhibition (5.4%).

The third anticancer drug identified as ClpP inhibitor in this study, bortezomib, inhibited the enzyme with an IC_50_ value in the nanomolar range and potently antagonized bovine α-chymotrypsin. No other compound structurally similar to bortezomib was included in the screened small molecule libraries. In mammalian cells, bortezomib acts as covalent and reversible inhibitor of the proteolytic activity of the 26S proteasome complex with high affinity and specificity [29]. Recently, it has been described as ClpP inhibitor in *Mycobacterium tuberculosis* [23]. Bortezomib is a dipeptidyl boronic acid and is chemically similar to the already known *E. coli* ClpP inhibitor Z-LY-CMK [21]. In agreement with literature data, bortezomib was confirmed as highly cytotoxic. As for the other two anticancer compounds, cisplatin and cDPCP, surface plasmon resonance studies revealed covalent binding of bortezomib to ClpP. Interestingly, in *E. coli* treated with the efflux pump substrate PaβN, bortezomib showed complete growth inhibition of the Δ*clpP* mutant, while the WT cell line growth was reduced to only 55%. This is possibly due to a combination of an off-target effect, which is unrelated to the inhibition of ClpP, and the clpP deletion.

3,4-DIC, is a general serine protease inhibitor targeting elastase-like, chymotrypsin-like, and trypsin-like proteases by acylation of the catalytic serine residue [30]. This explains the potent inhibition of both tested proteases in this study. As for bortezomib, no compounds structurally similar to 3,4-DIC were included in the screened small molecule libraries. 3,4-DIC revealed covalent binding to ClpP and did not show any intrinsic antimicrobial activities. In addition, the compound exhibited a limited effect on bacterial growth in the presence of nitric oxide stress.

Finally, we identified ClpP as a new intracellular target for cefmetazole, a second-generation cephalosporin. The enzyme inhibition assays revealed a low IC_50_ for *E. coli* ClpP and no inhibition of α-chymotrypsin. Moreover, the compound did not show cytotoxicity against human cell lines at therapeutically relevant concentrations. Cefmetazole interferes with transpeptidation and cross-linking during cell wall synthesis [27,31]. Covalent compound binding to penicillin-binding proteins involves a nucleophilic serine to attack the C–N bond of the β-lactam ring [27]. Therefore, a similar interaction with Ser97 of the active site appears to be the mechanism of action of cefmetazole in ClpP. Notably, the screened small molecule libraries in this study contained 43 additional cephems and penems and none of these compounds showed ClpP inhibition in the primary screen above >70%. Within the screened compound libraries, cefazolin, ceftriaxone, and cefotetan are structurally most similar to cefmetazole, as determined using a similarity search. However, none of these compounds revealed a significant ClpP inhibition in the primary screen (between −4.6% and 19.3%).

An explanation for the differences in enzyme inhibition might be the nitrile moiety in cefmetazole, which is not present in any other cephem or penem tested in this study. As nitrile groups may covalently react with amino acid side chains [32], cefmetazole may bind via two covalent bonds to ClpP. Molecular docking confirmed the possibility of two covalent bonds between the open β-lactam ring form of cefmetazole and Ser97, as well as Thr168 (via the nitrile moiety). In order to investigate this hypothesis, further experimental studies are required. In fact, the existence of two covalent bonds between a ClpP inhibitor and the enzyme has been shown in the X-ray crystal structure of ClpP in complex with Z-LY-CMK, involving Ser97 and His122 of the catalytic triad. A dual covalent binding mechanism of action might be exploited for the development of ClpP-specific inhibitors that may serve as adjuvants in antimicrobial therapies.

## 4. Materials and Methods 

### 4.1. Protein Production

Plasmid pETDclpPec (ORF ECK0431) [33] was inserted into *E. coli* SG1146a. After overnight incubation, bacteria were diluted in fresh LB medium containing 100 μg/mL ampicillin, and were grown until an OD_600_ of 0.6 was reached. Isopropyl β-D-1-thiogalactopyranoside (1 mM) was added for induction and flasks were placed in an orbital shaker for 5 h at 30 °C and 180 rpm. Cells were lysed on ice with a homogenizator (Precellys Evolution, Bertin Technologies, Montigny-le-Bretonneux, France). Protein was purified by immobilized metal affinity chromatography (IMAC) with a Ni-NTA His-tag resin column (Sigma Aldrich, Taufkirchen, Germany) using buffers with increasing concentration of imidazole (Buffer A: pH 7.6, 50 mM Tris-HCl buffer, 150 mM NaCl, 10 mM imidazole. Buffer B: pH 7.6, 50 mM Tris-HCl buffer, 150 mM NaCl, 20 mM imidazole. Buffer C: pH 7.6, 50 mM Tris-HCl buffer, 150 mM NaCl, 500 mM imidazole). Buffer exchange was carried out using PD 10 desalting columns (GE Healthcare, Little Chalfont, UK) with buffer D (pH 7.6, 20 mM Tris-HCl buffer, 100 mM NaCl, 5 mM MgCl, 10% (*v*/*v*) glycerol).

### 4.2. Primary Screening and Hit Confirmation

The SCREEN-WELL^®^ FDA-approved drug library V2 was purchased from Enzo Life Sciences, and the LOPAC^®1280^ library was obtained from Sigma-Aldrich (Taufkirchen, Germany). Moreover, a set of 329 MMP inhibitors was obtained from Specs (Zoetermeer, Netherlands). MMP inhibitors were dissolved at 10 mM concentration in 99.8% DMSO (ROTIPURAN^®^, Carl Roth GmbH, Karlsruhe, Germany) and stored at −20 °C until usage. Z-LY-CMK (Bachem, Bubendorf, Switzerland) was used as positive inhibition control.

The primary screen was carried out using a compound concentration of 200 μM. The proteolytic activity of ClpP in the presence of test compounds was assessed using a fluorescence-based assay in 384-well plate format (black, flat-bottom microtiter plates, #3820, Corning Inc., Corning, NY, USA), carried out on a Fluent^TM^ 780 laboratory automation system (Tecan, Männedorf, Switzerland). Briefly, 25 ng/μL of *E. coli* ClpP dissolved in a buffer containing 100 mM NaCl, 100 mM 4-(2-hydroxyethyl)-1-piperazineethanesulfonic acid (HEPES) pH 7.5, and 0.05% Brij^®^ 35 were transferred into each well. Test compound addition (final concentration 200 µM) was carried out using an Echo^®^ 550 liquid handling system (Labcyte, Sunnyvale, CA, USA). Protein and test compounds were incubated for 10 min at 30 °C, followed by addition of the fluorogenic substrate Suc-LY-AMC (75 μM, Enzo Life Sciences, Lörrach, Germany). Changes in fluorescence (excitation 350 nm, emission 435 nm) were monitored at 1 min intervals at 30 °C over 1 h using an Infinite M1000 microplate reader (Tecan).

For hit confirmation, 3,4-DIC (#D7910), cDPCP (#C0996) and cisplatin (#P4394) were obtained from Sigma-Aldrich (St. Louis, MO, USA), cefmetazole sodium (#KS-1415) and guanabenz acetate (#KS-1362) from Key Organics (UK), and bortezomib (#T2399) from TargetMol (Boston, MA, USA). All compounds were dissolved in DMSO (99.9%), resulting in 10 mM stock solutions. The assay was carried out as described above with increasing concentrations of test compounds. Dose-response experiments were done at least 3 times independently, each in technical triplicates. 

Compound selectivity was assessed using bovine α-chymotrypsin (#C4129, Sigma-Aldrich, St. Louis, MO, USA) at 200 µM. The assay was carried out as described above, except that 1 ng/μL of α-chymotrypsin and 100 μM of Suc-LY-AMC substrate were dissolved in a buffer containing 150 mM NaCl, 10 mM CaCl2, 50 mM Tris-HCl, and 0.05% Brij^®^ 35.

### 4.3. Surface Plasmon Resonance Spectroscopy

*E. coli* ClpP (80 μg/mL) protein was immobilized, using amine coupling (SPR-AS-HCA-03, Bruker Daltonics Inc., Billerica, MA, USA), onto the surface of the sensor chip of a SIERRA SPR-16 instrument (Bruker Daltonics GmbH, Bremen, Germany). ClpP protein was dissolved in acetate at pH 4. The running buffer contained 150 mM NaCl, 10 mM Hepes, 3 mM EDTA, and 0.05% (*v*/*v*) Tween-20. The flow rate for protein immobilization was 10 μL/min. Binding experiments were carried out using running buffer with the addition of 0.8% (*v*/*v*) of DMSO and 80 µM test substance (flow rate of 20 μL/min for 7 min and up to 30 min of dissociation time). The positive control for covalent binding was Z-LY-CMK, while caffeine was used as non-binding control. SPR data were analyzed using the Analyzer 3 software (Bruker Daltonics GmbH), which includes algorithms for subtracting reference signals and accounting for non-specific interactions between the solvent and the sensor chip surface.

### 4.4. Cell Viability Assays

Potential cell toxicity of the tested compounds was evaluated by ATP quantification using the CellTiter-Glo^®^ luminescent cell viability assay (Promega, Madison, WI, USA). A549, HTC-116 and MCF-7 cells were seeded at 2000 cells/well (20 µL) in white, flat-bottom, sterile 384-well plates (#781073, Greiner Bio-One GmbH, Frickenhausen, Germany) and incubated for 24 h. A total of 200 nL ClpP inhibitors plus positive (valinomycin, 10 µM) and negative (DMSO, 1%) controls were transferred in the plate, using an Echo^®^ 550 liquid handler (Labcyte) at 1:3 dilutions, starting from 200 μM, and incubated for an additional 48 h (95% air, 5% CO_2_ at 37 °C). After incubation, 20 µL of CellTiter-Glo^®^ reagent were added per well and incubated at room temperature in the dark for another 30 min. Luminescence was quantified by an EnVision multi-plate reader (PerkinElmer, Rodgau, Germany).

### 4.5. Antibacterial Assays

The potential of hit compounds to interfere with bacterial growth was evaluated in *E. coli* BW25-113 (wild type) and *tolC*-deficient strain *E. coli* JW5503 [34] (obtained from the NBRP-*E. coli* collection at the National Institute of Genetics, Mishima, Japan). The effect of the compounds on wild-type strain *E. coli* BW25-113 and the isogenic *clpP*-deficient *E. coli* JW0427-1 [34] (coli genetic stock center, New Haven, CT, USA) were measured in the presence of the efflux pump inhibitor Phe-Arg β-naphthylamide dihydrochloride (#P4157, Sigma-Aldrich, USA) at 25 μM. Single colonies of the bacterial strains from Mueller Hinton Agar culture were used to inoculate 5 mL of saline solution. The suspension was transferred into fresh, sterile Mueller Hinton broth (MHB) medium, resulting in a final concentration of 10^6^ CFU/mL. Growth assays were carried out in 96-well, flat-bottom, sterile clear plates (#167008, Nunc, VWR, Radnor, PA, USA) and compounds were tested at 50 and 100 μM concentration (2% DMSO). Absorbance at 600 nm was measured using the Multiskan GO or Varioskan LUX plate reader (Thermo Fisher Scientific, Vantaa, Finland) for 24 h at hourly intervals. Between measurements, plates were incubated in a shaking incubator (500 rpm) at 37 °C. 

Assessment of *E. coli* growth delay in presence of nitric oxide stress was carried out following our recently published protocol [24], which is an adaptation of the method reported by Robinson and Brynildsen [14] to a 96-well plate format. Fresh MHB medium was inoculated with isolated colonies of *E. coli* BW25-113 or *E. coli* JW0427-1, grown on Luria Agar, and left overnight in a shaking incubator (250 rpm) at 37 °C. On the following day, the overnight culture was used to inoculate fresh M9 medium (supplemented with 10 mM glucose) 1:100, and was grown until OD_600_ ~0.3. Afterwards, the culture was transferred into 96-well, flat-bottom, sterile clear plates (#167008, Nunc, VWR). Bacterial OD_600_ was adjusted to 0.1 with M9 medium (supplemented with 10 mM glucose). Test compounds were transferred into the 96-well plates, followed by addition of the bacterial culture. Nitric oxide stress was induced by addition of 2 mM DPTA NONOate ((Z)-1-[N-(3-aminopropyl)-N-(3-ammoniopropyl)amino]diazen-1-ium-1,2-diolate, Cayman Chemical, Ann Arbor, MI, USA), dissolved in NaOH (final concentration 0.14 mM). Absorbance measurements (600 nm) were done with a Multiskan GO or Varioskan LUX plate reader (Thermo Fisher Scientific, Finland) at hourly intervals for 24 h. Between measurements, plates were incubated in a shaking incubator (500 rpm) at 37 °C.

### 4.6. Data Analysis

Calculation of Z prime (Z’) for HTS assay performance was done according to Zhang et al. [25]. Plates were considered for further analyses if Z’ > 0.6 [26]. IC_50_ values were determined by a non-linear fit using the equation for sigmoidal dose-response with variable slope in GraphPad Prism 7.03 (GraphPad software, La Jolla, CA, USA).

### 4.7. Molecular Docking and Similarity Search

The ClpP X-ray crystal structure was obtained from the Protein Data Bank (PDB ID 2ZFS). Of the 14 chains, chain B was selected based on structural quality analysis of all chains using PROCHECK [35] and ProSa [36]. The protein structure was prepared using the software Molecular Operating Environment (MOE, Chemical Computing Group Inc., Montreal, QC, Canada) version 2016.0802. Three-dimensional coordinates of cefmetazole were generated within MOE. Molecular docking was carried out using GOLD version 5.6.1 (Cambridge Crystallographic Data Centre, Cambridge, UK) and the GoldScore scoring function. GoldScore revealed the best redocking performance of all four implemented scoring functions when redocking the co-crystallized ligand Z-LY-CMK in terms of root-mean-square deviation between ligand crystal coordinates and the top-ranked docking pose. The search space was defined as a sphere of radius 15 Å centered on atom OG of Ser97. Altogether, 50 docking runs were conducted. The early termination option was disabled and the side chain of Asn150 was defined as flexible.

The similarity search was carried out using MOE. For all compounds in the screened compound libraries, molecular fingerprints (Bit-Packed MACCS structural keys) were calculated. A similarity threshold of 70% was applied for searching compounds structurally similar to the confirmed primary hits.

## Figures and Tables

**Table 1:** Overview of IC_50_ values of identified ClpP inhibitors and the positive control (Z-LY-CMK), confidence interval (CI) as well as inhibition of *E. coli* ClpP and bovine α-chymotrypsin at 200 μM compound concentration.

Compound	*E. coli* ClpP		Inhibition (%) at 200 μM	
	IC_50_ (μM)	CI	*E. coli* ClpP	α-Chymotrypsin
Bortezomib	0.04	0.02–0.05	100.1	97.8
Cefmetazole	3.4	1.5–7.6	94.5	0
cDPCP	24.6	17–36	91.2	84.7
Cisplatin	31	21.4–44.8	96.5/98.7 ^1^	0
3,4-DIC	4.9	3.3–7.2	98.9	101.4
Z-LY-CMK	14	11.0–16.6	98.6	99.8

^1^ Cisplatin emerged as hit in both screened libraries of approved and investigational drugs.

**Table 2:** Cytotoxicity potential of ClpP inhibitors against the human cell lines HTC-116, MCF-7, and A549 as quantified by a luminescent cell viability assay (IC_50_). The ratio compares the results with ClpP inhibition as shown in Table 1 (IC_50_ cytotoxicity/IC_50_ ClpP).

Compound	Cytotoxicity					
	HTC-116		MCF-7		A549	
	IC_50_ (µM)	Ratio	IC_50_ (µM)	Ratio	IC_50_ (µM)	Ratio
Bortezomib	0.0002	0.005	0.04	1	0.0002	0.005
Cefmetazole	5430	1597.06	74.12	21.8	250	73.53
cDPCP	283	11.5	3.27	0.13	131	5.33
Cisplatin	913	29.45	4.52	0.15	86.7	2.8
3,4-DIC	45.1	9.2	9.06	1.85	44.7	9.12
Z-LY-CMK	3.54	0.25	0.05	0	7.15	0.51

**Table 3:** Antibacterial evaluation of the ClpP inhibitors. All compounds were tested at 100 μM concentration and growth inhibition was measured after 24 h of incubation at 37 °C.

Strain	PAβN	Compound	Growth Inhibition (%)
*E. coli* WT	-	Cefmetazole	100
	+	Cefmetazole	100
*E. coli* Δ*clpP*	-	Cefmetazole	100
	+	Cefmetazole	100
*E. coli* Δ*tolC*	-	Cefmetazole	100
*E. coli* WT	+	Bortezomib	55
*E. coli* Δ*clpP*	+	Bortezomib	100

## Supplementary Materials

Supplementary materials can be found at https://www.mdpi.com/1422-0067/20/11/2686/s1.

## Abbreviations

CI	Confidence interval
ClpP	Caseinolytic protease subunit P
HTS	High-throughput screening
MMP	Matrix metalloproteinase
Z-LY-CMK	benzyloxycarbonyl-leucyl-tyrosine chloromethyl ketone
3,4-DIC	3,4-dichloroisocoumarin
