# Supplementary material for: Identification and Characterization of Approved Drugs and Drug-Like Compounds as Covalent Escherichia coli ClpP Inhibitors
Source: Int J Mol Sci. 2019 May 31;20(11):2686. doi: 10.3390/ijms20112686 (PMC6600665; doi:10.3390/ijms20112686)
Supplement: Supplementary file 1 [file ijms-20-02686-s001.pdf]

Supplementary Material

Identification and characterization of approved drugs and drug-like compounds as covalent *Escherichia coli* ClpP inhibitors

Elisa Sassetti[1,2] Cristina D. Cruz[3], Päivi Tammela[3], Mathias Winterhalter[2], Koen Augustyns[4], Philip Gribbon[1], Björn Windshügel,[1,*]

[1]Fraunhofer Institute for Molecular Biology and Applied Ecology IME, ScreeningPort, Hamburg, Germany

[2] Department of Life Sciences and Chemistry, Jacobs University Bremen gGmbH, Bremen, Germany

[3] Drug Research Program, Division of Pharmaceutical Biosciences, University of Helsinki, Helsinki, Finland

[4] Laboratory of Medicinal Chemistry, University of Antwerp, Antwerp, Belgium

***Correspondence:**
Dr. Björn Windshügel,
bjoern.windshuegel@ime.fraunhofer.de

Supplementary Figures

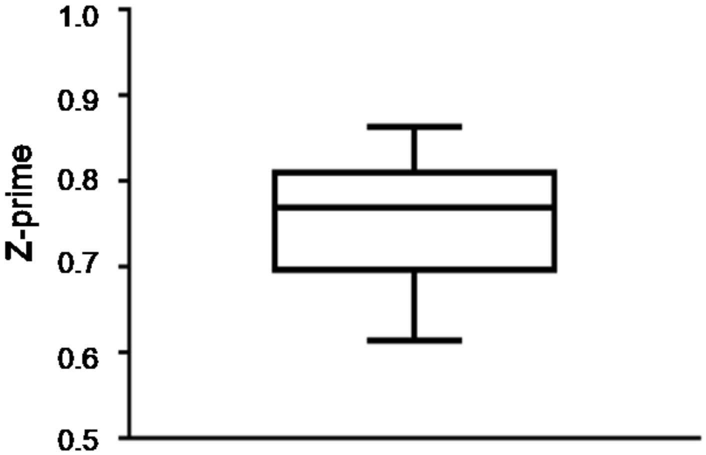

Figure S1. Box plot of the Z’ (Z-prime) calculated for the high-throughput screen.

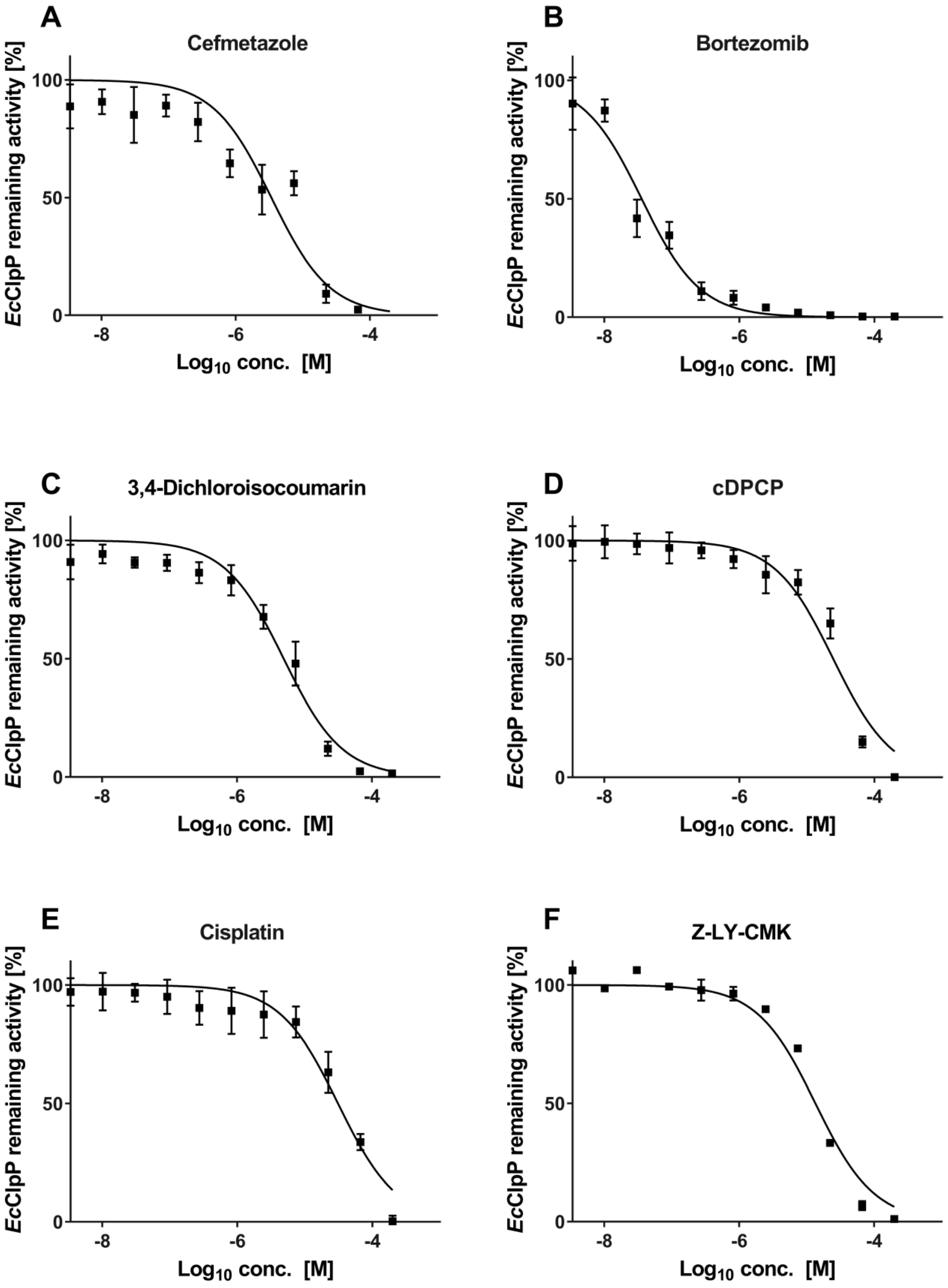

Figure S2. Dose-response curves (1:3 dilution starting from 200 μM) for hit compounds (A-E) selected after high-throughput screening and the known inhibitor Z-LY-CMK (F). Error bars represent biological triplicates with three internal replicates each.

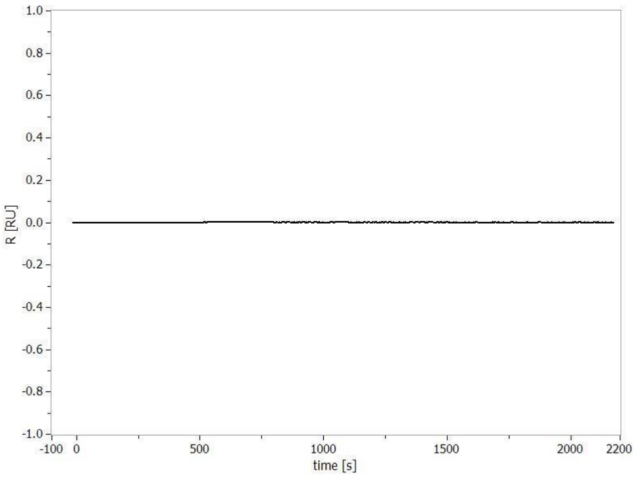

Figure S3. SPR sensorgram of the negative control caffeine at 80 µM concentration.

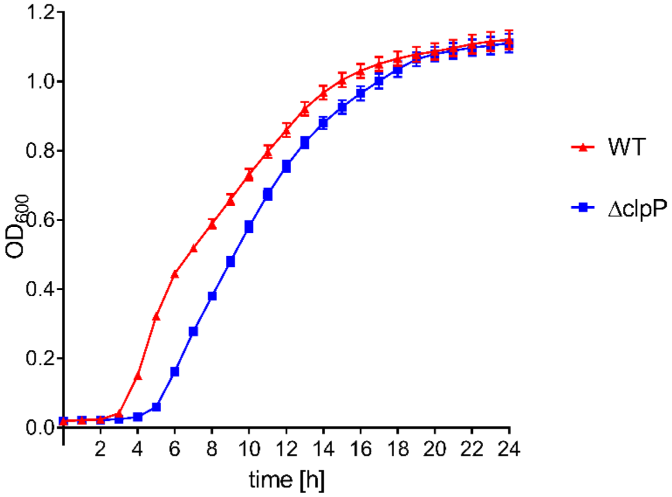

Figure S4. Bacterial growth assays for WT (red) and Δ*clpP* (blue) *E. coli* strains in rich media (MHB) over 24 hours. Each value represents the mean of three independent experiments ± standard deviation.

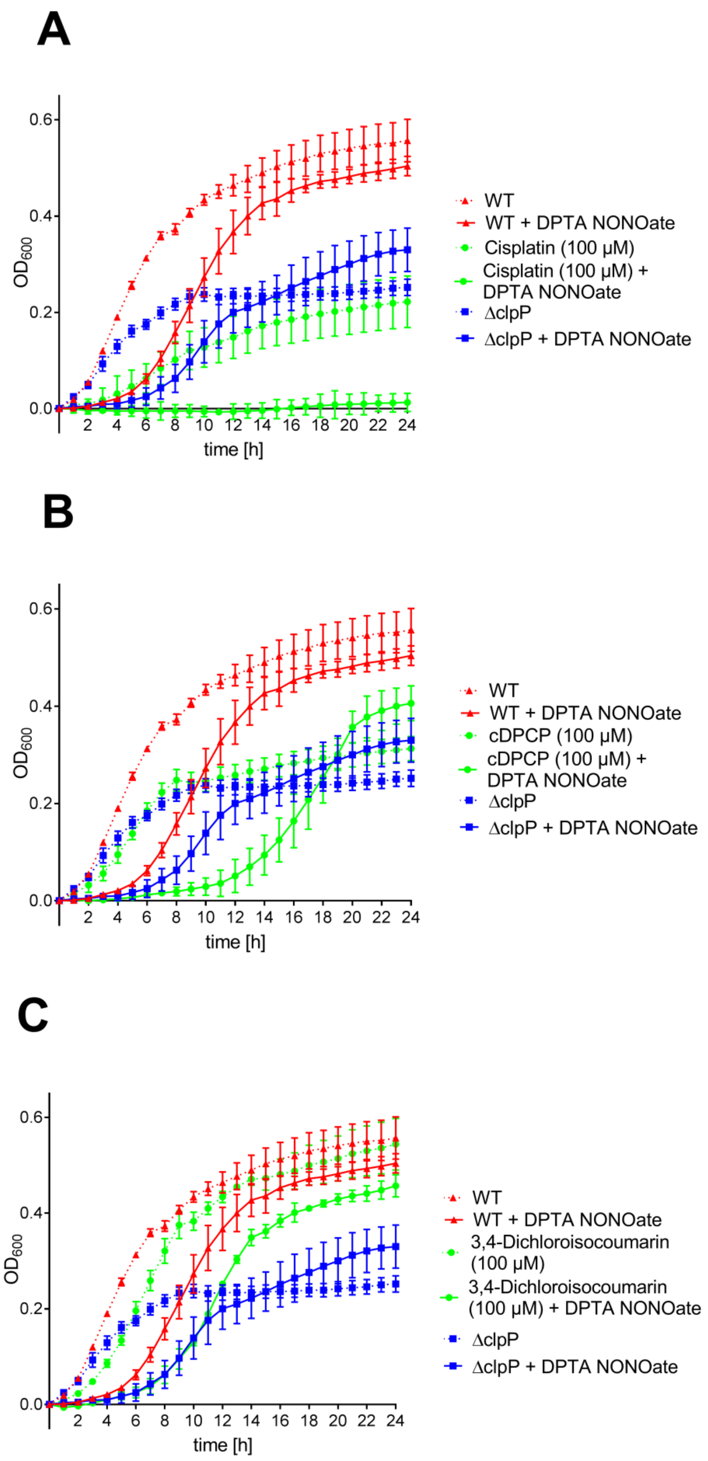

Figure S5. Bacterial growth curves of WT and Δ*clpP E. coli* strains in minimal media, in presence (solid lines) and absence (dotted lines) of DPTA NONOate (NO·) induced stress for (A) Cisplatin, (B) cDPCP and (C) 3,4-DIC at 100 µM compound concentration. Each value represents the mean of three independent experiment ± standard deviation.

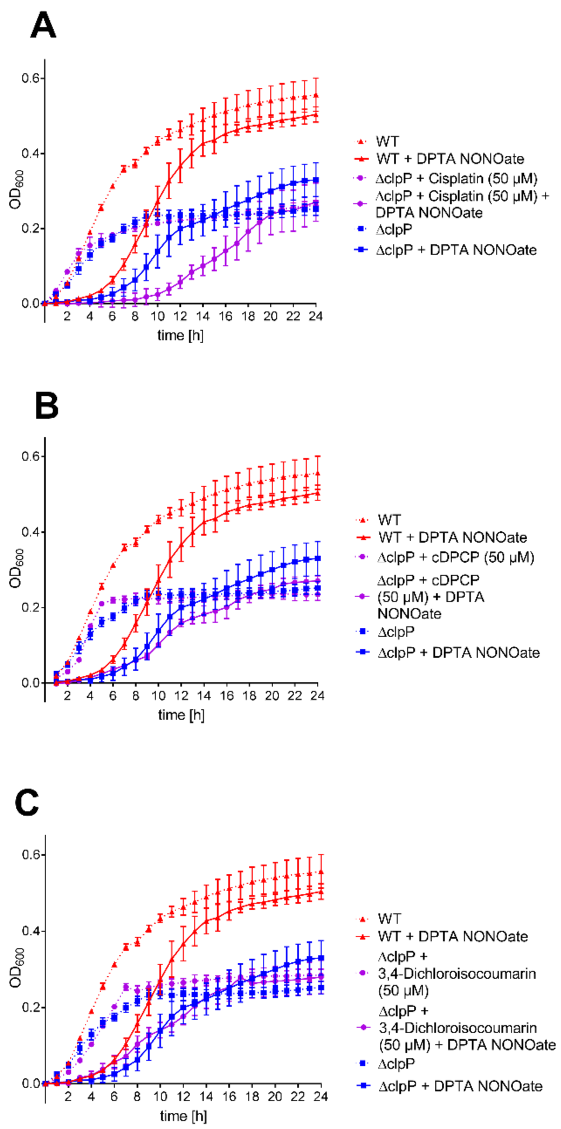

Figure S6. Bacterial growth curves of WT and ΔclpP *E. coli* strains in minimal media and in presence (solid lines) and absence (dotted lines) of DTPA NONOate (NO•). OD600 was measured in absence and presence of 50 µM (A) cisplatin (B) cDPCP and (C) 3,4-DIC. Each value represents the mean of three independent experiment ± standard deviation.

Supplementary Tables

Table S1. List of the compounds with <70 % remaining activity of *E. coli* ClpP (200 µM) in the primary screen. Cisplatin was present in two of the screened libraries resulting as hit in both.

Compound name	Library	CAS	*E. coli* ClpP inhibition [%]
Bortezomib	SCREEN-WELL®	179324-69-7	100.00
Atazanavir	SCREEN-WELL®	198904-31-3	85.03
Sulfasalazine	SCREEN-WELL®	599-79-1	77.33
Balsalazide	SCREEN-WELL®	80573-04-2	76.46
Rifapentine	SCREEN-WELL®	61379-65-5	71.54
Silver Sulfadiazine	SCREEN-WELL®	22199-08-2	88.38
Sunitinib Malate	SCREEN-WELL®	341031-54-7	80.67
Nitazoxanide	SCREEN-WELL®	55981-09-4	79.96
Cisplatin	SCREEN-WELL®, LOPAC®1280	15663-27-1	96.54<br>98.67
Ezatiostat	LOPAC®1280	168682-53-9	88.80
Cefmetazole sodium	LOPAC®1280	56796-39-5	94.51
10058-F4	LOPAC®1280	403811-55-2	72.82
Guanabenz acetate	LOPAC®1280	23256-50-0	100.00
Bisdemethoxycurcumin	LOPAC®1280	33171-05-0	76.54
SR 27897 hydrate	LOPAC®1280	136381-85-6	84.71

S 24795	LOPAC®1280	304679-75-2	76.27
3, 4-Dichloroisocoumarin	LOPAC®1280	51050-59-0	98.92
Retinoic acid p-hydroxyanilide	LOPAC®1280	65646-68-6	74.12
Myricetin	LOPAC®1280	529-44-2	76.04
Tyrphostin 51	LOPAC®1280	126433-07-6	72.80
Tyrphostin 23	LOPAC®1280	118409-57-7	79.28
cDPCP	LOPAC®1280	106343-54-8	91.16
2-({4-[(cyclohexylamino)sulfonyl]anilino}carbonyl)cyclohexanecarboxylic acid	MMP- Specs	n.a.	72.93
3-cyclopentyl-N-[2-(4-morpholinyl)ethyl]propanamide	MMP- Specs	n.a.	84.44

Table S2. Complete list of cephems and penems compounds in the screened compound libraries.

**Compound name**	**Library**	**CAS**
Cefepime hydrochloride Hydrate	SCREEN-WELL®	123171-59-5
Dicloxacillin sodium Salt Monohydrate	SCREEN-WELL®	13412-64-1
Doripenem	SCREEN-WELL®	148016-81-3

Imipenem	SCREEN-WELL®	64221-86-9
Orlistat (Tetrahydrolipstatin)	SCREEN-WELL®	96829-58-2
Meropenem	SCREEN-WELL®	96036-03-2
Ampicillin Trihydrate	SCREEN-WELL®	7177-48-2
Aztreonam	SCREEN-WELL®	78110-38-0
Ceftazidime	SCREEN-WELL®	72558-82-8
Oxacillin sodium salt monohydrate	SCREEN-WELL®	7240-38-2
Penicillin V Potassium	SCREEN-WELL®	132-98-9
Piperacillin	SCREEN-WELL®	61477-96-1
Amoxicillin	SCREEN-WELL®	26787-78-0
Cefadroxil	SCREEN-WELL®	66592-87-8
Cefdinir	SCREEN-WELL®	91832-40-5
Cefditoren Pivoxil	SCREEN-WELL®	117467-28-4
Cefixime	SCREEN-WELL®	79350-37-1
Cefotetan Disodium	SCREEN-WELL®	74356-00-6
Cefotaxime Acid	SCREEN-WELL®	63527-52-6
Cefpodoxime Proxetil	SCREEN-WELL®	87239-81-4
Cefprozil	SCREEN-WELL®	92665-29-7
Ceftibuten	SCREEN-WELL®	97519-39-6
Ceftizoxim sodium	SCREEN-WELL®	68401-82-1

Cefuroxime Axetil	SCREEN-WELL®	64544-07-6
Cefuroxime sodium	SCREEN-WELL®	56238-63-2
Cephalexin Monohydrate	SCREEN-WELL®	23325-78-2
Clavulanate Potassium	SCREEN-WELL®	61177-45-5
Cloxacillin sodium	SCREEN-WELL®	7081-44-9
Ezetimibe	SCREEN-WELL®	163222-33-1
Nafcillin sodium	SCREEN-WELL®	985-16-0
Penicillin G Potassium (Benzylpenicillin)	SCREEN-WELL®	113-98-4
Cefaclor	SCREEN-WELL®, LOPAC®1280	53994-73-3
Cefazolin sodium	SCREEN-WELL®, LOPAC®1280	27164-46-1
Ceftriaxone sodium	SCREEN-WELL®, LOPAC®1280	104376-79-6
Cephalexin hydrate	LOPAC®1280	15686-71-2
Cefsulodin sodium salt hydrate	LOPAC®1280	52152-93-9
Cefmetazole sodium	LOPAC®1280	56796-39-5
Imipenem monohydrate	LOPAC®1280	74431-23-5
Cephalosporin C zinc salt	LOPAC®1280	59143-60-1
Cephalothin sodium	LOPAC®1280	58-71-9
Cephradine	LOPAC®1280	38821-53-3
Cefotaxime sodium	LOPAC®1280	64485-93-4

Pivmecillinam	LOPAC®1280	32886-97-8
N-cyclohexyl-1-(2-oxo-1-azetidinyl)cyclohexanecarboxamide	MMP-Spec	n.a.
N-cyclohexyl-1-(2-oxo-1-azetidinyl)cyclopentanecarboxamide	MMP-Spec	n.a.
